# Rationale, design and conduct of a school-based anti–smoking intervention: the “PEPITES” cluster randomized trial

**DOI:** 10.1186/s12889-018-5840-8

**Published:** 2018-07-31

**Authors:** Stéphanie Vieira, Fabrice Chéruel, Hélène Sancho-Garnier

**Affiliations:** 1 Fondation JDB Prévention Cancer, 2-4 rue du Mont Louvet, 91 640, Fontenay-Lès-Briis, France; 2 Université Paris-Sud, Université Paris-Saclay, 91405 Orsay cedex, France; 3 Institut du Cancer de Montpellier (ICM), Parc Euromédecine, 34298 Montpellier cedex, France

**Keywords:** Health education, Secondary school, Long term anti-smoking interventions, Cluster randomized trial

## Abstract

**Background:**

In France smoking initiation rates amongst 11 to 16 year-olds are worryingly high. Several studies show that early initiation to psycho-active substances is a strong predictor of tobacco addiction. Decreasing the age at which tobacco use starts represents a key challenge for reducing tobacco usage. Implementing an intervention trial using educational workshops based on the Theory of Planned Behavior (TPB) and covering the 4 years of secondary school could be effective.

**Methods:**

“PEPITES” is an interventional research, using a cluster randomized design. It will allow assessing the effectiveness of interventions both in reducing the tobacco initiation rate and the regular smoking rate of secondary school pupils. We will also evaluate the process of the implementation of the study and thus will help to the transferability of the intervention.

A partnership convention was signed between the JDB Foundation and the National Education authority which designated 6 secondary state schools for the PEPITES trial.

The 6 schools were randomly allocated to 3 groups of 2 clusters each: 1 control group, 2 different intervention groups with 2 workshops per year during 4 years; In one of this group the 2 last workshops will be dedicated to measure the loss of taste due to tobacco smoking. In each school, all pupils in year 1 with a signed parental authorization (744 pupils) have been included in the trial. The interventions targets one of the variables of the TPB and the reinforcement of psycho-social competencies. We estimated that we could detect a reduction of increase ≥5.5 and 8**%** respectively in the 2 principal outcomes (risk α of 5%, and β of 80%).

**Discussion:**

Carrying out a randomized prevention trial in the school environment raises specific problems which it seems useful to detail for other educational actors who would like to perform a similar study.

This discussion concerns the acceptation and cooperation of the National Education partners, the risks of contamination, the information given to parents and pupils and their consent, and the representativeness of the schools involved.

**Trial registration:**

ISRCTN85812512. Registered 15 May 2018 by BioMed Central. (retrospectively registered).

## Background

In France smoking initiation rates amongst 11 to 16 year-olds continue to be worryingly high, with however some positive indicators in the Ile-de-France Paris region (IDF) compared to the rest of the French mainland [1]. With the age of first tobacco smoking between 12 and 14 years old and the age of daily tobacco use between 14 and 16 years old, it is clear that young people who have not tried tobacco are in the minority at high school: in IDF at the age of 17, near 63% have already tried tobacco and 27% smoke every day [2]; in the Essonne department (IDF) at the age of 17, 23% were smoking every day in 2011 [3]. If, in the early 2000s, tobacco usage was falling, the numbers have since leveled off, as demonstrated by the 2011 and 2014 Escapad surveys [4]. In this context, the French Social Affairs Ministry launched a national policy to try to reduce the number of people smoking daily to below 20% by 2024 [5]. The means to achieve this reduction focus in particular on actions directed at younger people. In France, until now, few actions have been effective in pushing back the age at which tobacco is first consumed and in reducing tobacco addiction amongst young people.

Many international programs, which aim to reduce tobacco consumption in young people, are carried out in the school environment, and, by doing so, they are able to address a more or less «captive audience» as well as to monitor the actions and outcomes over a period of several years [6, 7]. The approaches which have been identified as promising involve putting in place a tobacco prevention pathway throughout the entire school years with various annual sessions incorporated in the school program and conducted by professionals from outside the school. These sessions consist of workshops based on behavioral modulator theories and on the increase of the psychosocial competencies of the school children [8] support from parents and school personnel (« zero cigarette » policy in school and outside school, help to stop smoking) might increase the effectiveness of tobacco prevention actions amongst young children [9].

Several studies show that early initiation to psycho-active substances (tobacco, cannabis, alcohol) is a strong predictor of tobacco addiction [10]. Early initiation leads to stronger addiction and a lower ability to stop smoking [10]. Signs of nicotine dependence appear right from the first weeks of consumption by young people [11].

Increasing the age at which tobacco use starts represents a key challenge for reducing tobacco usage by the population later in their lives . A study carried out in the French Languedoc-Roussillon region showed that there are two key moments in the evolution of smoking amongst youngster: initiation between 12 and 13 years old (5th and 4^th^school levels) and established habit between 15 and 16 years old (3rd and 2nd levels) which leads to addiction [12]*.*

Furthermore it has been clearly demonstrated that, in order for prevention actions to be effective, they need to be repeated regularly; one action alone, outside of the usual environment, has little chance of changing the behavior of individuals in the long term.

Considering all of the above points, the present intervention trial on tobacco prevention covering the 4 years of secondary school (11 to 15 years old) was implemented in the Essonne department where the JDB Foundation for Cancer Prevention is based. This trial is based on a cluster randomization trial design comparing 3 groups: two intervention groups and a control group, thus allowing a better evaluation of the effectiveness of the interventions.

Within the scope of the proposed educational workshops based on the theory of planned behaviors (TPB) [13, 14]; we also test the hypothesis that the concrete and measurable discovery by pupils of sensory modifications rapidly provoked by tobacco, such as loss of taste, might lead to a better understanding of the harmful effects of tobacco. This might increase the motivation of young people to not start smoking or to stop consuming. This paper describe the protocol of the PEPITES trial (**P**rogram in **E**ssonne for the **P**revention of the **I**nitiation to **T**obacco through **E**ducation in the **S**chool environment), and its implementation in 6 secondary schools in the Essonne department.

## Methods

### Aims and study design

PEPITES is an interventional research study in primary prevention, using the cluster randomized trial design. This methodology will allow assessing the effectiveness of the interventions both in reducing the tobacco initiation rate and the regular smoking rate of secondary school pupils. We will also evaluate the process of the implementation of the study *(*what works, for whom, for what and in what circumstances) and thus will help to the transferability of the intervention [15].

Randomized cluster is used where individual randomization is not ethically possible: all pupils in the same class, or of the same level of class of a given secondary school, must enjoy the same supposed beneficial activities for their health*.* Additionally this technique avoids contamination, that is that randomized pupils in the control arm be influenced by pupils from the intervention arm and change their behavior spontaneously [16] .

This research protocol has been validated by the Consultative Committee for Information Processing in relation to Research in the Health field (CCTIRS) previously to obtain the mandatory CNIL (Commission Informatique et des Libertés) authorization and by the Ethical Evaluation Committee of INSERM (Institut National de la Santé et de la Recherche Médicale).

### Population selection and randomization

A partnership convention was signed between the JDB Foundation and the Essonne National education authority which designated 6 secondary mixed sex state schools for the PEPITES trial as part of their commitment to health education.

After this designation and before randomization, school head teachers were sent detailed information of the study protocol, followed by a visit by study team members. Then a member of the school management was designated to be the key contact for the study investigators.

The randomization process was carried out by a study investigator during a meeting organized at the JDB Foundation where representatives of each secondary school were present.

**Fig. 1:**
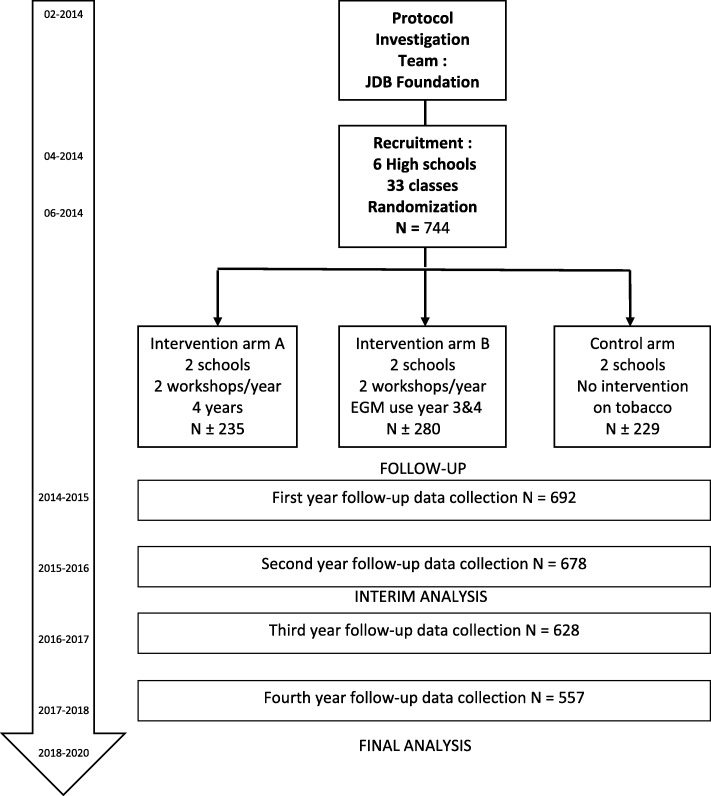
PEPITES trial design

The 6 schools were allocated to 3 groups of 2 clusters each (Fig. 1):

- one group of 2 secondary schools with 2 educational workshops per school year; that is 8 interventions per secondary school over the 4 years,
- one group of 2 secondary schools with 2 educational workshops per school year; that is 8 interventions per secondary school over the 4 years, with one of the workshops dedicated to measure taste with the electrogustometer during the 3rd and 4th years,

All the educational workshops were carried out by the JDB Foundation professional prevention trainee;

- one control group of 2 secondary schools with no intervention planned by the JDB Foundation.

### Study population

In each secondary school, all pupils in year 1 (grade 6) at the time of the survey, with a signed parental authorization form (94% of year 1 pupils), have been included in the PEPITES trial.

Written parental authorization was requested for the whole duration of the trial after sending a letter which outlined the principal objectives of the trial and the intervention undertaken. The pupils could also refuse to participate even if the parents had given their authorization.

At the start of the study in September 2014, the included population was 744 pupils covering 33 classes in the 6 secondary schools and were expected to be followed, during the 4 years of secondary school.

### Proposed educational interventions

The proposed interventions targets one of the variables of the TPB (i.e., attitude, subjective and social norms, perceived control and intention) and the reinforcement of psycho-social competencies (resistance to pressure, critical mind, reasoned choice, civil responsibility). Teaching methods are based on inter-activity and practical experimentation.

**Fig. 2:**
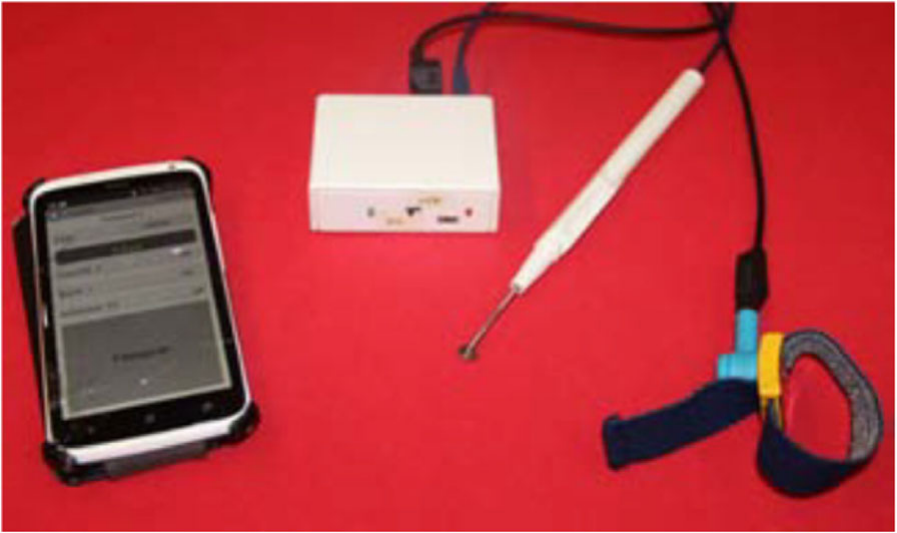
Electrogustometer (Unpublished figure: created by the authors for this manuscript)

- ➢ The educational sessions are adapted to the age of the pupils, tested and conducted by prevention professionals from the JDB Foundation. They are carried out during school time and last 45 min. In chronological order they cover the following themes: reasons for starting smoking, awareness of the risks taken, explanation of the marketing strategies of the tobacco industry, the mechanism of addiction and the effects on health (Table 1).
- ➢ Experimental session measuring taste change linked to tobacco use. Our hypothesis is that the concrete consciousness of the harm caused by tobacco use could increase children awareness regarding the harmful effects of tobacco and consequently improve their motivation to not start smoking or to stop if they have started. A tool, the electrogustometer (Fig. 2), makes it possible to measure the taste sensitivity of the tongue - sensitivity which reduces significantly for a smoker from the first cigarette regularly smoked [17].

**Table 1:** Content of educational sessions

Year 1 (Age ± 11): Why do people start smoking?	
ᅟThink about the reasons for starting smoking and be aware of the space tobacco occupies in our society today (Peer pressure).	
ᅟAt the end of the workshop, the pupils should be able to: ᅟᅟ− Think about what influences a person to start smoking (or not) ᅟᅟ− Expose the major lines of the history of tobacco in our society	
Year 2 (Age ± 12): Risk behavior, Stress management, Risk taking and alternatives	
ᅟThink about what risks they take in their daily lives	
ᅟAt the end of the workshop, the pupils should be able to: ᅟᅟ− Explain what constitutes risk behavior ᅟᅟ− Imagine alternatives to risk taking	
Year 3 (Age ± 13): Manipulation by the tobacco industry – Marketing strategy	
ᅟDevelop a critical mind by analyzing sales techniques and influencing techniques of the tobacco industry	
ᅟAt the end of the workshop, the pupils should be able to: ᅟᅟ• Discuss the main determinants in the regular consumption of tobacco ᅟᅟ• Quote the marketing strategies used by the tobacco industry	
Year 4 (Age ± 14): Tobacco: Effects and addiction	
ᅟReinforce knowledge on addiction	
ᅟAt the end of the workshop, the pupils should be able to: ᅟUnderstand the link of dependence between a smoker and his tobacco ᅟDescribe the effects of tobacco on the body organs.

In the second intervention group, during years 3 and 4, one educational session is dedicated to the use of the electrogustometer, and to observe results of pupils who are smokers or non-smokers.

### Outcomes measures

#### Evaluation of the results

- The 2 principal outcomes for the PEPITES trial are:
  - The prevalence of pupils having experimented (at least once) tobacco smoking at the end of year 2 and year 4 as compared to beginning of year 1 (comparison of the 2 intervention groups together versus the control group)
  - The prevalence of regular (at least weekly) cigarette smokers at the end of year 4 (comparisons of the 3 groups 2 by 2).
- These outcomes will be collected at the beginning of each year and at the end of year 2 and 4, and their evolution over time analyzed in each of the groups and compared. Outcomes prevalence measures are only based on pupils declaration.
- The secondary outcomes include experimentation with hookah, cannabis, electronic cigarettes, perception of norms, attitudes and future intentions of the participants with regard to smoking.

The various determinants which may have a role in starting smoking or becoming a regular smoker (gender, siblings, sporting activity, home area (ZUS), smokers in the entourage (parents, friends…) will also be studied and reported.

#### Evaluation of the process

The detailed evaluation of the process aims to study the context of the implementation of PEPITES in the school environment by identifying the external factors which might have an impact on the implementation (levers and obstacles) and on its effectiveness (such as: how well the rule forbidding smoking in the school is applied, other tobacco sessions carried out in the control schools, national campaigns, etc.). The aim of this evaluation is to be able, if the results are positive, to transfer these interventions in other secondary schools. If the results are inconclusive, such an analysis might help to explain the reasons.

### Power of the study

The number of pupils that could be included in the trial is around 750 split into 3 groups of the same size: one control group (T), 2 intervention groups: (A) without EGM experimentation and (B) with EGM use in years 3 and 4. We calculated the rate of reduction of increase in the 2 principal outcomes we could detect, with a global risk α of 5%, a risk β of 20% and an intra-cluster correlation coefficient of “starters and regular smokers” at base line of 0,020 (one-sided test).

1. Comparison Control (T) versus Interventions (A + B) at the end of year 2 on the reduction in increase of *“tobacco use initiation*” (where the increase rate in Essonne is around 10% at the end of the 2 first years of secondary school), and considering that the intervention group A + B (N1) will included ±500 pupils and the control group T (N2) ± 250 (N1/N2 = ½), a significant reduction **≥5.5%** could be detected**.**
2. For the «regular smokers» criterion (where the percentage is estimated at 5% in Essonne at the end of year 2 and 10% in year 4), a significant reduction ≥**4%** at the end of year 2 and **≥ 6.5%** at the end of year 4 could be detected .
3. Comparison 2 by 2 of each of the 3 groups (a control group and 2 intervention groups), for the final analysis at the end of year 4, we will be able to demonstrate a significant reduction increase **≥10%** of “starters” (from the present 25% rate) and a reduction **≥8%** of regular smokers (from the present 10% rate) .

Taking into account in the analysis the factors linked to these 2 outcomes and using appropriate multiple regression analysis and multi-level modeling should allow for an increase in power.

In order to reduce loss of power, the number of pupils lost to follow-up (final questionnaire not filled in at the end of year 2 or year 4) will be minimized by actively searching for them if absent at the time of completing the questionnaire or through the organization of catch-up educational workshops whenever possible.

### The organization of the trial

The implementation of such a complex intervention program requires detailed preparation prior to the start of the trial. After randomization, a steering committee was set up to identify all the necessary steps to ensure the successful implementation of PEPITES.

A presentation to the teaching staff and the school management team was made by the scientific manager and a professional prevention trainer from the JDB Foundation before the trial started.

In each secondary school, key contact person was nominated (school nurse or Principal Education Advisor) so as to ensure coordination with the JDB Foundation team. This contact person was responsible for overseeing the overall organization of the interventions: draw up a planning, reserve the classrooms, inform the teachers and pupils concerned, distribute an information letter to the parents and collect their consent forms.

### Data collection

The procedures and tools for data collection were created by the JDB Foundation team and were tested before the implementation of the trial in various secondary schools not included in the study.

Regarding the process, qualitative methods (semi-directive interviews and completion of a log-book notifying absences, refusals, computers problem as well as comments relating to the data collection) were put in place.

Questionnaires were used to identify reasons for parents’ refusals and to collect the opinions of the main participants (teaching staff, management team).

Regarding the pupils whose parents had given their authorization and who agreed to participate, the answers to the questions were collected by means of computerized and anonymous self-assessment questionnaires (Table 2), completed in class during school time. The conditions for completion of the questionnaire were to be alone at the computer under the supervision of two adults per class to avoid any copying or minimize reporting bias and to help if necessary.

**Table 2:** Guarantee of anonymity

The self-assessment questionnaire contains a code which allows the answers from the same pupil to be followed from year to year. This numbered code was randomly generated and assigned to each pupil in the trial. The code is recorded in a list with the pupils’ names which is held by the headmaster of the school in his role as a reliable third party: the headmaster does not have access to the pupils’ answers; the JDB Foundation team which performs the analysis does not have access to the list of pupils’ names. Each time a new questionnaire is completed the code is again supplied to the pupil thanks to the paper list held by the headmaster of the school. This information is also included in the information letter to parents and pupils before completion. The procedures put in place have met that the National Commission for Data Protection and Liberties (CNIL -France) requirements and has authorized the implementation of the PEPITES trial.	

The pupils were clearly informed that neither the teachers, nor their parents, nor anybody else could match their name to their answers. Pupils were also informed that they had the right to refuse to take part.

The questionnaires are completed at different times:

- for the intervention groups: T0 at the start of each school year; T1 and T2 after each educational session; T3 at the end of year 2; and T4 at the end of year 4.
- in the control schools, data was collected at T0, T3 and T4.

The questionnaires T0, T3 and T4 cover the various determinants which may play a role in starting smoking and in becoming a regular smoker. The same basic questions (T0) are used at each follow-up so as to provide data with comparable results, whereas other questions are included (T3 and T4) to explore questions of secondary research interest.

The questionnaires T0, T3, T4 have 2 sections:

- one describing some personal and socio-demographic characteristics of the pupil, his family surroundings with regard to passive smoking, his knowledge and beliefs regarding smoking, and his future intentions regarding consuming tobacco,
- the other describing his behavior with regard to tobacco use that is: experimenter, regular or occasional smoker, ex-smoker, non-smoker. The questions concerning smoking are those used in surveys carried out at the national level in France [18] and in Europe [19].

The questionnaires T1 and T2 evaluate the acquisition of knowledge and the modifications of behavioral determinants linked to the workshops. For the educational sessions which measure taste, the data measured by electrogustometer is not collected.

Logical checks for pertinent questions are carried out by the data entry system. .

### Statistical analysis

Data analysis will follow a pre-specified plan including:

- ➢ The initial description of the study population and the comparison between the 3 groups. Observed differences which are linked to smoking, should lead to an adjustment when the groups are compared;
- ➢ Follow-up of pupils over time: carrying out the planned workshops, number of ‘lost’ pupils, how well the questionnaires are filled in, modifications of the collected characteristics and behaviors;
- ➢ Searching for factors which influence smoking;
- ➢ Comparison of the 2 principal outcomes at the end of year 2 (2 groups) and at the end of year 4 (2 and 3 groups) and the time trends over 4 years of such outcomes. Taking into account the cluster effect, matched data, the 2 analysis of the trial and any relevant adjustment factors, (different logistical regression models adapted to the types of data will be studied).

For all these analyses, the software STATA/SE version 13 will be used.

## Discussion

Carrying out, in France, a randomized intervention trial on health prevention risks in the school environment raises a certain number of specific problems which it would seem useful to detail for other educational actors who would like to carry out a similar complex study.

This discussion concerns the acceptation, the interest and the cooperation of the National Education partners, the minimization of the risks of contamination, information given to parents and pupils and their consent, ensuring that collected data remains anonymous, representativeness of the schools that take part….

### Agreement of partner schools to participate

First of all it is necessary to convince the regional Head of the National Education (Rectorat) of the interest of the project and especially of the methodology of the study allowing an « evidence based » evaluation which is a vital element to allow a possible transferability to other schools.

The search for schools with which to collaborate relies on several key principles [20] which are: clarity of information, understanding and acceptance of the methodology by partners and parents, minimization of the work load for the school staff, a planned and reactive organization and regular contact between all parties.

The identification of a key contact person for each secondary school (often the school nurse as the subject of the trial is health) makes it easier to carry out a rigorous study. The specific context (public or private, social environment…) of each school must be taken into consideration as much as possible.

### Contamination

The possibility, for the control group, to be influenced by the intervention group is unavoidable if the random selection process is carried out on an individual basis, which leads to the pupils in the compared groups spending time and communicating with each other and possibly copying each other, which can reduce the effect of the interventions and thus the possibility of it being identified. Furthermore it would be unethical not to propose actions which aim to improve health to all the pupils of the same class.

To avoid this phenomenon, the cluster randomization design will determine at random the group allocated to each participating school. With this methodology all of the pupils from one school belong to the same group: either intervention, or control. Agreement of schools to participate occurs before randomization which can leave certain schools frustrated if designated as a control group and they may feel aggrieved; consequently they may try to compensate by involving other preventive actions, which may of course incur a major bias to the trial. It is vital that the control schools understand the importance of their “passive” participation and to propose other educational activities which do not interfere with the trial’s objective. The cluster design may also leads to an increase in the variability of the outcomes and consequently a loss of power which must be compensated for by increasing the number of subjects included in the trial.

### Consent, anonymity and data collection

As well as the agreement of the teaching staff it is also necessary to obtain the agreement of the parents and pupils.

In France, the parental written consent form is mandatory for minors (legal obligation) and to obtain it is the responsibility of the headmaster. Written information, or oral information for the parents, is compulsory. A letter summarizing the main objectives of the survey, as well as details of the interventions, has to be sent to parents at least 2 or 3 weeks before the trial began. The parents are therefore able to notify their refusal.

- The pupils, authorized by their parents to participate, agreed to participate by the fact they filled in the questionnaire that they are given. The pupils could refuse to participate on the first day of the trial without the need for any justification and with no penalty for their school record. However, if we want to have a participation level close to 100% and honest answers, the pupils must have understood on the one hand the objective of the trial and on the other hand the fact that the answers are anonymous. They must be reassured that it is impossible for their answers to be communicated to their parents or teachers. A clear and full explanation on this point is all the more important since the questions relate to acts which, for them, are not allowed. The message to the pupils must be friendly, confident and adapted to their age group.

Use of electronic tablets to fill in the questionnaires is greatly appreciated by the pupils and as a result constitutes a user-friendly tool which is very useful for data collection. If there are technical problems (computer breakdown, no internet connection), questionnaires in paper format should be ready to be used.

The procedures for filling in the questionnaire must be explained at the beginning of the session by the study team. The pupils are invited to ask questions throughout the session if they do not understand a question. The teachers are asked to identify the pupils who have difficulty reading or concentrating and who may need extra help to be able to participate. Once the students have completed their questionnaires, the data must be sent directly to a secure server.

### ‘Lost’ pupils, reactive organization

Planning the actions in advance allows developing a good working relationship with the school staff and they are key contacts for the study team throughout the trial. These relationships are maintained through regular contact, at least 2 meetings by school year regarding the trial’s progress and the carrying out of all the study activities, are planned and agreed with the schools (to avoid disturbances to their timetables).

In order to maximize participation, the study team can return to each school around two weeks after the initial data collection so that the data of pupils who were absent can be collected.

### Representativeness and transferability

One solution for a trial covering a population of pupils, which would be representative of the entire school territory studied, would be the possibility of randomly selecting the participating schools and that all the schools agree to participate… but such a process would require several months of dialogue with all the schools and remains somewhat unrealistic.

Consequently it will remain to assess, if the results of the randomized trial are positive, whether these results can be extrapolated to another context: that is the key challenge of the transferability studies which will have to follow the demonstration of efficacy of a preventive intervention before being widely generalized. A detailed evaluation of the process of the initial trial is of primary importance in order to perform such a transferability study. The Functions /Implementation /Context model [21] could contribute to improve the description of the process of the initial trial and facilitate to acquire the knowledge of the factors involved in its transferability.

## Conclusion

The implementation and follow-up over 4 years of such a study will allow us, first, to better identify the levers and obstacles related to the implementation of a controlled intervention trial in the school environment and, secondary, to organize the transfer of effective interventions in a more general context.

## Abbreviations

CCTIRS: Comité Consultatif sur le Traitement de l’Information en matière de Recherche dans le domaine de la Santé
CNIL: Commission Nationale de l’Informatique et des Libertés
DESDEN: Direction Education des Services Départementaux de l’Education Nationale
EGM: Electrogustometer
IDF: Ile-de-France
INSERM: Institut National de la Santé et de la Recherche Médicale
JDB: Justin De Bouville
PEPITES: Programme Essonnien de Prévention de l’Initiation au Tabac par l’Education en milieu Scolaire / Program in Essonne for the Prevention of the Initiation to Tobacco through Education in the School environment
TPB: Theory of Planned Behavior
ZUS: Zone Urbaine Sensible
